# Automated Multistep Parameter Identification of SPMSMs in Large-Scale Applications Using Cloud Computing Resources

**DOI:** 10.3390/s21144699

**Published:** 2021-07-09

**Authors:** Elia Brescia, Donatello Costantino, Federico Marzo, Paolo Roberto Massenio, Giuseppe Leonardo Cascella, David Naso

**Affiliations:** Department of Electrical Engineering and Information Technology, Politecnico di Bari, 70126 Bari, Italy; donatello.costantino@poliba.it (D.C.); f.marzo1@studenti.poliba.it (F.M.); paoloroberto.massenio@poliba.it (P.R.M.); giuseppeleonardo.cascella@poliba.it (G.L.C.); david.naso@poliba.it (D.N.)

**Keywords:** adaline neural network, cloud computing, internet of things, parameter identification, permanent magnet synchronous machines, R-statistic, steady-state identification

## Abstract

Parameter identification of permanent magnet synchronous machines (PMSMs) represents a well-established research area. However, parameter estimation of multiple running machines in large-scale applications has not yet been investigated. In this context, a flexible and automated approach is required to minimize complexity, costs, and human interventions without requiring machine information. This paper proposes a novel identification strategy for surface PMSMs (SPMSMs), highly suitable for large-scale systems. A novel multistep approach using measurement data at different operating conditions of the SPMSM is proposed to perform the parameter identification without requiring signal injection, extra sensors, machine information, and human interventions. Thus, the proposed method overcomes numerous issues of the existing parameter identification schemes. An IoT/cloud architecture is designed to implement the proposed multistep procedure and massively perform SPMSM parameter identifications. Finally, hardware-in-the-loop results show the effectiveness of the proposed approach.

## 1. Introduction

Permanent magnet synchronous machines (PMSMs) are widely employed in several applications such as industrial servo drives [1], electric vehicles [2], wind power generators [3,4], and aeronautical systems [5]. To enhance performances while predicting faults and maintenance operations, parameter identification of PMSMs represents a well-established research area [6]. The PMSM parameter identification problem can be stated as follows: once voltages, currents, and speed measurements are available, find the winding dq-axis inductances, resistance, and rotor flux linkage [6,7,8,9,10]. Simultaneous PMSM multiparametric estimation leads to rank-deficient problems, i.e., the number of unknown parameters exceeds the rank of the system, causing large estimation errors [6,7,8]. To overcome such an issue, many approaches have been proposed.

A simple method to obtain a full-rank identification problem is based on fixing some parameters to their nominal values, as in [11,12]. However, such methodology is compromised by mismatches occurring between nominal and actual values [7,8]. Moreover, those methods are not practical for large-scale systems where some nominal parameters may be unknown. Another approach is based on the use of extra sensors, such as thermal sensors and power or torque meters [13,14]. This approach is not suitable in large-scale applications due to the increased complexity and costs [7]. 

Full-rank identification problems can also be obtained by means of online signal injection-based algorithms implemented via adaline neural networks (AdNNs) [8,9], RLS procedures [15,16], or particle swarm optimization (PSO) [17]. Offline multiparametric identifications are provided in [18] via voltage injection and in [19] via an amplitude-auto-adjusting d-axis current injection. The rotor position offset is used as a perturbating signal for the rotor flux linkage and stator resistance estimations in [10]. Although they are effective, signal injection-based methods require accessing and programming the control unit of a PMSM, which is clearly not practical in large-scale industrial processes. An alternative approach to solving the rank-deficiency problem is based on the use of measurement data at different operating conditions of the machine. This approach has been adopted in [20,21] using the least square (LS) algorithm, but it cannot be used with zero d-axis current controllers due to the resulting non-invertibility of the LS data matrix. Nevertheless, no information, additional sensors, or signal injections are needed, making such a method suitable for large-scale applications. However, the estimation accuracy is jeopardized by the parameter variations occurring when the operating condition changes [7]. Moreover, this method cannot detect parameter variations during the motor operations to improve control and monitoring performances. 

To the authors’ best knowledge, the parameter estimation of multiple running machines in large-scale applications has not yet been investigated. To minimize complexity, costs, and human interventions without requiring machine information, a flexible and automated approach is required. The aim of this paper is to overcome the limitations of the existing parameter identification techniques by proposing a novel identification strategy for surface PMSMs (SPMSM), highly suitable for large-scale systems. A novel multistep approach using measurement data at different operating conditions of the SPMSM is proposed to solve the rank deficiency problem. Three AdNNs with mutual updating based on the R-statistic algorithm are employed to separately identify the stator resistance, stator inductance, and rotor flux linkage. Moreover, a cloud architecture implements the proposed algorithm providing an effective and flexible large-scale identification scheme. Cloud computing is an Industry 4.0 key technology ([22]) which enables an easy deployment of computational-demanding algorithms to deal with the parameter estimation problem of large-scale systems. Few works have addressed the parameter identification in cloud environments. In [23], the Microsoft Azure public cloud is employed to solve parameter estimation problems in computational systems biology. In [24], a multi-objective PSO implements the parameter identification of a soil model in a cloud environment. In [25], an offline parameter identification of an electric vehicle traction battery is performed using cloud computing resources. In [26], a model reference adaptive system is implemented for the automated parameter identification of the rotor flux linkage of SPMSMs using an AWS-based cloud prototype. However, note that, compared to [26], the present work deals with the identification of all the electrical parameters of SPMSMs and does not require the knowledge of the nominal values.

Main features and novelties of this paper are as follows:

- We solve the rank-deficient problem by employing a multistep procedure based on three AdNNs with mutual updating without using signal injection as in [8,9,10,15,16,17,18,19];
- Our method does not require exact knowledge of nominal values, overcoming the issues of [11,12];
- Extra sensors are not required as in [13,14];
- Unlike [20,21], the proposed method also handles zero d-axis current control schemes;
- An automated solution based on the R-statistic algorithm allows one to properly identify the SPMSM steady-state operating conditions in which the multistep procedure operates;
- Low computational requirements on the edge-side make the proposed method highly suitable for IoT integrations, without any change on the motor control unit;
- Off-the-shelf cloud technologies implement a hardware-in-the-loop (HIL) setup while simulation results show the effectiveness of the proposed approach.

Compared to the existing solutions, we point out how the main advantage of the proposed method is its applicability to large-scale industrial processes due to an automated identification scheme and the limitation of human intervention.

This paper is organized as follows. In Section 2 the rank-deficiency problem is discussed. The proposed algorithm is detailed in Section 3. Section 4 describes the HIL setup used for validation, while Section 5 presents the simulation results. Finally, concluding remarks are reported in Section 6. 

## 2. Rank-Deficiency Problem

We consider a field-oriented control (FOC) of an SPMSM drive. The dynamical model of an SPMSM in the dq rotating synchronous reference frame is described by the following equations [3]:

(1) $$v_{d}\;=R_{s}i_{d}+L_{s}\frac{di_{d}}{dt}-\omega_{r}L_{s}i_{q},$$

(2) $$v_{q}\;=\;R_{s}i_{q}+L_{s}\frac{di_{q}}{dt}+\omega_{r}L_{s}i_{d}+\omega_{r}\psi ,$$

where $v_{d}$, $v_{q}$ are the dq-axes voltages, $R_{s}$ and $L_{s}$ are the stator resistance and dq-axes inductance, respectively, $i_{d}$, $i_{q}$ are the dq-axes currents, $\omega_{r}$ is the electrical rotor speed, and ψ is the rotor flux linkage. Note that if a zero d-axis current control is performed, at the steady state, (1) and (2) can be rewritten in the following form:

(3) $$v_{d}\;=\omega_{r}L_{s}i_{q},$$

(4) $$v_{q}\;=\;R_{s}i_{q}+\omega_{r}\psi .$$

From (3), $L_{s}$ can be estimated independently from $R_{s}$ and ψ through the d-axis voltage, speed, and q-axis current measurements. Instead, $R_{s}$ and ψ cannot be estimated simultaneously from (4), since only one equation is available. This is commonly known as rank deficiency problem for the parameter identification of SPMSMs controlled with $i_{d}=0$. In this paper, a multistep procedure is proposed to solve such a problem.

## 3. Novel Multistep Parameter Identification Algorithm Based on Adaline NNs

**Figure 1:**
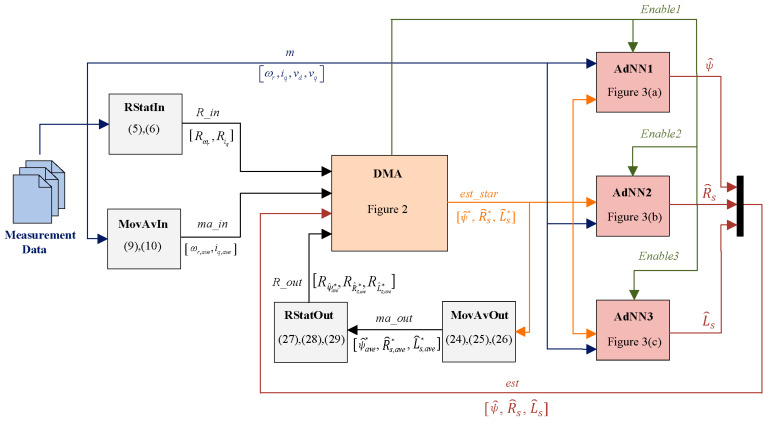
Scheme of the parameter identification algorithm.

The proposed identification scheme is shown in Figure 1. Starting from the measurement data, the RStatIn block recognizes the SPMSM steady-state conditions, the MovAvIn filters the measurement data, the decision-making algorithm (DMA) rules the operations of the three AdNNs, one per parameter: rotor flux linkage (AdNN1), stator resistance (AdNN2), and inductance (AdNN3). RStatOut detects the steady state of the parameter estimations which are filtered by the MovAvOut. These blocks are detailed in the following subsections.

### 3.1. Measurement Data

The measurement data contain the samples of $i_{q}$, $\omega_{r}$, $v_{d},$ and $v_{q}$, which are gathered in signal m in Figure 1. It is assumed that the d-axis current is zero to perform a maximum torque per ampere control and that no additional signals are injected for the purpose of the parameter identification.

### 3.2. RStatIn: Identificator of Steady-State Conditions of the SPMSM

R-statistic is a statistical method developed to automatically distinguish transient states from steady states in noisy processes [27]. The RStatIn block employs the R-statistic method for the automated identification of the steady-state conditions of the SPMSM. This operation is required since the AdNN estimators operate only during the steady-state conditions of the motor, as will be detailed below. The SPMSM operates in steady-state conditions when its speed and electromagnetic torque are constant. Therefore, to identify the steady-state conditions of the SPMSM, the R-statistic is applied on the electrical rotor speed and q-axis current.

The R-statistic procedure calculates the following indices [27]:

(5) $$R_{\omega_{r}}\left(k\right)=2\frac{\sum_{j=k-N}^{k}\omega_{r,n}{\left(j\right)}^{2}-\frac{1}{N}{\left(\sum_{j=k-N}^{k}\omega_{r,n}\left(j\right)\right)}^{2}}{\sum_{i=k-N+1}^{k}{\left(\omega_{r,n}\left(j\right)-\omega_{r,n}\left(j-1\right)\right)}^{2}},$$

(6) $$R_{i_{q}}\left(k\right)=2\frac{\sum_{j=k-N}^{k}i_{q,n}{\left(j\right)}^{2}-\frac{1}{N}{\left(\sum_{j=k-N}^{k}i_{q,n}\left(j\right)\right)}^{2}}{\sum_{j=k-N+1}^{k}{\left(i_{q,n}\left(j\right)-i_{q,n}\left(j-1\right)\right)}^{2}}.$$

where $R_{\omega_{r}}$ and $R_{i_{q}}$ are the indices for $\omega_{r}$ and $i_{q}$, respectively, k is the k-th sample processed, and N is the sample’s window length. $R_{\omega_{r}}$ and $R_{i_{q}}$ are gathered in signal R_in in Figure 1. $\omega_{r,n}$ and $i_{q,n}$ are computed as follows:

(7) $$\omega_{r,n}\left(k\right)=\omega_{r}\left(k\right)+w_{\omega_{r}}\left(k\right),\;\;w_{\omega_{r}}\left(k\right)=\sigma_{\omega_{r}}\left(k\right)\sqrt{-2ln\left(r_{1}\left(k\right)\right)}sin\left(2\pi r_{2}\left(k\right)\right),$$

(8) $$i_{q,n}\left(k\right)=i_{q}\left(k\right)+w_{i_{q}}\left(k\right),\;\;w_{i_{q}}\left(k\right)=\sigma_{i_{q}}\left(k\right)\sqrt{-2ln\left(r_{1}\left(k\right)\right)}sin\left(2\pi r_{2}\left(k\right)\right).$$

where $w_{\omega_{r}}$, $w_{i_{q}}$ are noise signals based on the Box–Muller method, $\sigma_{\omega_{r}}$ and $\sigma_{i_{q}}$ are the standard deviations, while $r_{1}$ and $r_{2}$ are independent samples chosen from the uniform distribution in the interval [0, 1]. The noise signals are introduced to avoid numerical issues [27].

$R_{\omega_{r}}$ and $\;R_{i_{q}}$ tend to be around 1 as the process tends to the steady state. Instead, during transients, the indices are expected to be greater than 1. Hence, the process is in the steady state if $R\leq R_{crt}$, where $R_{crt}$ is a critical threshold. In [27], the author suggests manually tuning $R_{crt}$ according to the actual responses. 

$R_{crt}$, N, $\sigma_{\omega_{r}}$, and $\sigma_{i_{q}}$ are the tuning parameters of RStatIn found with trial-and-error approach. The tuning of these parameters does not need to be repeated for different electrical drives since they do not depend upon physical parameters of the machine. Note that the R-statistic algorithm acts as a filter on the sample window of length N, hence it is affected by delay in identifying the beginning or the end of a steady state. This issue has been considered and properly compensated through to the MovAvOut block, as will be shown below.

### 3.3. MovAvIn: Moving Average of Measurement Data

The MovAvIn block is used to filter the input signals $\omega_{r}$ and $i_{q}$ as follows:

(9) $$\omega_{r,ave}\left(k\right)=\frac{1}{N}\overset{k}{\underset{j=k-N}{\sum }}\omega_{r}\left(j\right),$$

(10) $$i_{q,ave}\left(k\right)=\frac{1}{N}\overset{k}{\underset{j=k-N}{\sum }}i_{q}\left(j\right),$$

where $\omega_{r,ave}$ and $i_{q,ave}$ are the moving averages (gathered in signal *ma_in* in Figure 1). Note that the windows size, N, is the same as in RStatIn. The outputs of MovAvIn are considered as the current steady-state operating conditions of the SPMSM by the DMA. 

### 3.4. Decision-Making Algorithm

The DMA enables the AdNNs through the Enable signals to execute the multistep parameter identification. AdNN1 and AdNN2 are enabled in different operating conditions while AdNN3 works simultaneously with AdNN1 or AdNN2. The DMA employs the information provided by the RStatIn block, $R\_in=[R_{\omega_{r}},\;R_{i_{q}}]$, the current operating conditions from MovAvIn, $ma\_in=[\omega_{r,ave},\;i_{q,ave}],$ and a convergence condition to decide which of the two estimators (AdNN1 or AdNN2) should be enabled. Moreover, the DMA employs information provided by the RStatOut block, $R\_out=\left[R_{{\hat{\psi }}_{ave}^{*}},R_{{\hat{R}}_{s,ave}^{*}},\;R_{{\hat{L}}_{s,ave}^{*}}\right]$, to accept only the filtered parameter estimations,$\;ma\_out=\left[{\hat{\psi }}_{ave}^{*},\;{\hat{R}}_{s,ave}^{*},\;{\hat{L}}_{s,ave}^{*}\right]$, which reach the steady state. Finally, the DMA performs a stop criterion which automatically concludes the multistep procedure without human intervention. The DMA outputs are the Enable signals for the three AdNNs and $est\_star=[{\hat{\psi }}^{*}$, ${\hat{R}}_{s}^{*}$, ${\hat{L}}_{s}^{*}]$. Moreover, the DMA uses three internal states, i.e., ${\hat{\psi }}_{stop}\left(x\right)$,$\;{\hat{R}}_{stop}(y$), and ${\hat{L}}_{stop}\left(z\right)$, called partial estimations, to compute ${\hat{\psi }}^{*}$, ${\hat{R}}_{s}^{*}$, ${\hat{L}}_{s}^{*}$ when the AdNNs are not active; also, they are used in the stop criterion. Three indices, x, y, z are updated asynchronously as explained later. Finally, the DMA employs other internal states, called check variables, which are $\omega_{rR}\left(y\right)$, $\omega_{r\psi }\left(x\right)$*,* $i_{qR}\left(y\right)$, and $i_{q\psi }\left(x\right)$, representing respectively the speed and q-axis current at which the latest rotor flux linkage and stator resistance estimations have been performed by the AdNNs. All these variables are initialized as follows: the three Enable signals are set to OFF;$\;{\hat{\psi }}^{*}$, ${\hat{R}}_{s}^{*}$, ${\hat{L}}_{s}^{*}$, ${\hat{\psi }}_{stop}$,$\;{\hat{R}}_{stop}$, ${\hat{L}}_{stop}$, x, y, and z are set to zero; $\;\omega_{rR}$ and $i_{qR}$ are set to M and ε, respectively, where M is a large number and ε is a small number; $\omega_{r\psi }$ and $i_{q\psi }$ are set to zero.

**Figure 2:**
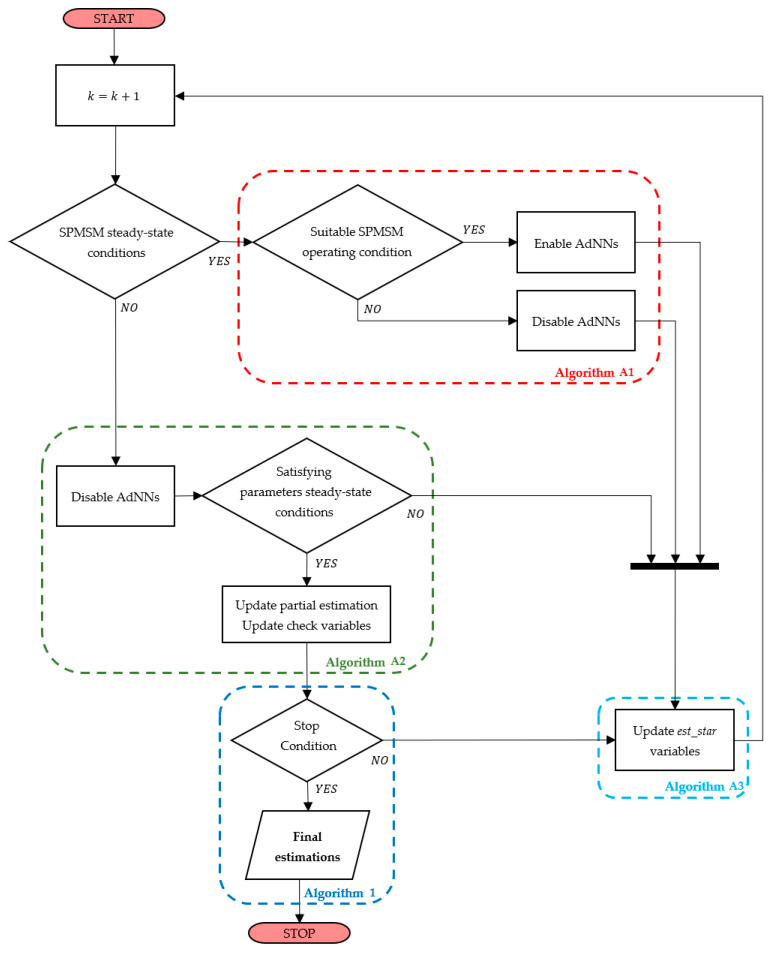
Flow chart of the proposed DMA.

The operation of the DMA is described in the flow chart in Figure 2 At each iteration, the DMA checks if the SPMSM is in the steady-state condition. If the SPMSM is in the steady state, i.e., $R_{\omega_{r}}\left(k\right)\leq R_{crt}$ and $R_{i_{q}}\left(k\right)\leq R_{crt}$, the DMA implements Algorithm A1, providing the first parameter estimation by activating AdNN1 and AdNN3 if x=0. The first estimation initializes the multistep procedure, obtaining the first partial estimations ${\hat{\psi }}_{stop}$ and ${\hat{L}}_{stop}$. Once the first estimation ${\hat{\psi }}_{stop}$ has been achieved, the SPMSM stationary operating conditions are inspected. As proven in Appendix B, to make sure that stator resistance and rotor flux linkage estimation errors asymptotically converge to zero, the AdNN1 and AdNN2 must be activated separately to satisfy the following convergence condition (A13):

(11) $$\frac{i_{q\psi }}{i_{qR}}\cdot \frac{\omega_{rR}}{\omega_{r\psi }}<1,$$

where $i_{q\psi },$ $\omega_{r\psi }$, $i_{qR},$ and $\omega_{rR}$ are the check variables defined above, which express the operating conditions of the SPMSM in which the AdNN1 and AdNN2 are enabled by the DMA. In order to satisfy (11), the DMA activates the AdNN1 if $\left(\omega_{rR}/(\omega_{r,ave}\left(k\right)\right)\cdot \left(i_{q,ave}\left(k\right)/i_{qR}\right)<0.95$ and activates the AdNN2 if $(\omega_{r,ave}\left(k\right)/\omega_{r\psi })\cdot \left(i_{q\psi }/i_{q,ave}\left(k\right)\right)<0.95$. The AdNN3 is activated together with AdNN1 or AdNN2 since its performances are not affected by the operating conditions of the SPMSM, as shown in Section 2. If the current SPMSM operating conditions do not satisfy the above inequalities, all the enable signals are set to OFF. 

If $R_{\omega_{r}}\left(k\right)>R_{crt}$ or $R_{i_{q}}\left(k\right)>R_{crt}$, the DMA performs the operations reported in Algorithm A2. Firstly, the Enable signals are set to OFF, since the AdNNs should work only during steady-state operations of the SPMSM. Then, if the AdNNs were active and their filtered estimations were at the steady state in the previous step, i.e., k−1, the DMA updates ${\hat{\psi }}_{stop}$,$\;{\hat{R}}_{stop},$ and ${\hat{L}}_{stop}$ with the values in ma_out(k−1) and $\omega_{rR}$, $\omega_{r\psi }$, $i_{qR}$, and $i_{q\psi }$ with the values in ma_in(k−1). After the updating of the partial estimations, the DMA checks the stop criterion, which is described in Algorithm 1. This algorithm operates on the sets of the last $N_{stop}$ samples of ${\hat{\psi }}_{stop}$, ${\hat{R}}_{stop},$ and ${\hat{L}}_{stop}$ where $\varepsilon_{stop}$ is an arbitrary small value. If the AdNNs were not active or the provided estimations were not in the steady state in the previous step, ${\hat{\psi }}_{stop}$,$\;{\hat{R}}_{stop}$, ${\hat{L}}_{stop}$, $\omega_{rR}$, $\omega_{r\psi }$, $i_{qR}$, and $i_{q\psi }$ are not updated. This allows one to accept only the stationary estimations provided by the AdNNs, avoiding the introduction of estimation errors due to transients of the AdNNs. Note that, if the AdNNs estimations are accepted, the partial estimations assume the values of the filtered estimations provided by the MovAvOut block. The filtering reduces the errors introduced by the estimations performed during transients. In fact, as stated in Section 3.2, the R-statistic algorithm detects with a delay the loss of the steady state of the SPMSM. During this delay, the AdNNs continue to produce estimations which are affected by unwanted perturbations that must be filtered. Finally, the DMA updates the output variables, est_star, according to Algorithm A3. Note that if the AdNNs are activated, the est_star variables are set to the corresponding AdNNs estimations; otherwise, they are set to the partial estimations. 

We remark how the partial estimations are updated asynchronously and only if the corresponding ma_out variables have reached their steady states, as described in Algorithm A2. Moreover, the partial estimations represent the results of the proposed algorithm, since they are the only estimations not affected by errors due to the transient states.
**Algorithm 1.** Stop criterion.$A=\left\{{\hat{\psi }}_{stop}\left(x-N_{stop}+1\right),\;{\hat{\psi }}_{stop}\left(x-N_{stop}+2\right),\ldots ,\;{\hat{\psi }}_{stop}\left(x\right)\right\}$$B=\left\{{\hat{R}}_{stop}\left(y-N_{stop}+1\right),\;{\hat{R}}_{stop}\left(y-N_{stop}+2\right),\ldots ,\;{\hat{R}}_{stop}\left(y\right)\right\}$$C=\left\{{\hat{L}}_{stop}\left(z-N_{stop}+1\right),\;{\hat{L}}_{stop}\left(z-N_{stop}+2\right),\ldots ,\;{\hat{L}}_{stop}\left(z\right)\right\}$$if\;\;\frac{\min\left(A\right)-\min\left(A\setminus \min\left(A\right)\right)}{\min\left(A\right)}<\varepsilon_{stop}\;\;AND\;\frac{\min\left(B\right)-\min\left(B\setminus \min\left(B\right)\right)}{\min\left(B\right)}<\varepsilon_{stop}\;AND\;\frac{\min\left(C\right)-\min\left(C\setminus \min\left(C\right)\right)}{\min\left(C\right)}<\varepsilon_{stop}$${\hat{\psi }}_{stop}\left(x+1\right)=\min\left(A\right),\;\;{\hat{R}}_{stop}\left(y+1\right)=\min\left(B\right),{\hat{L}}_{stop}\left(z+1\right)=\min\left(C\right)$

### 3.5. Adaline NNs

**Figure 3:**
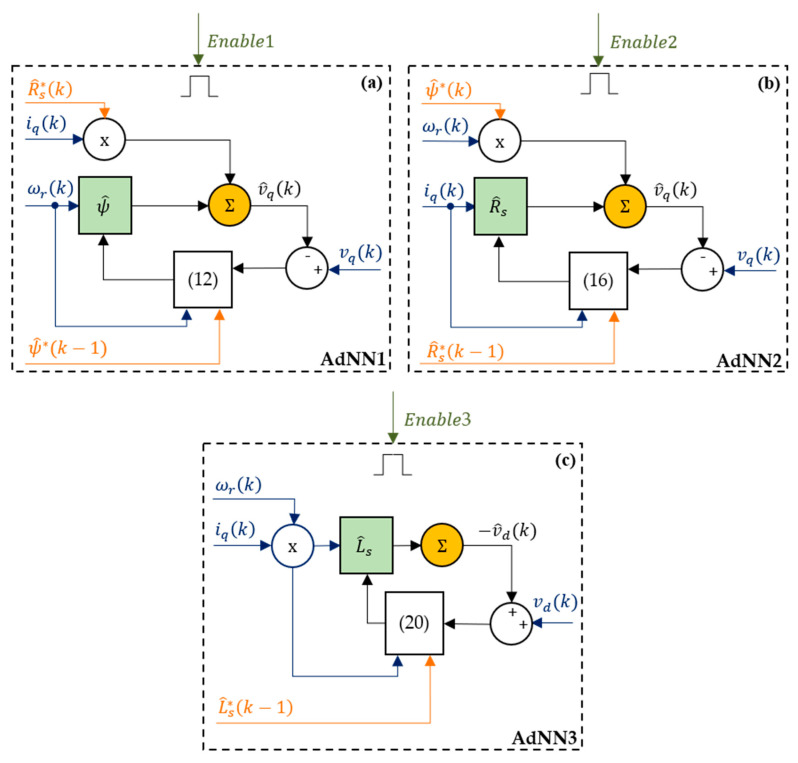
Structures of the Adaline NNs: (**a**) rotor flux linkage estimator; (**b**) stator resistance estimator; (**c**) stator inductance estimator.

This section describes the neural network estimators implemented in this work and depicted in Figure 3. These estimators are driven by the Enable signals provided by the DMA. The AdNN1 receives as inputs the measurements of the q-axis current,$\;i_{q}$, the electrical rotor speed,$\;\omega_{r}$, and the q-axis voltage, $v_{q}$, and the estimations ${\hat{\psi }}^{*}$ and ${\hat{R}}_{s}^{*}$ provided by the DMA. The weight of the electrical rotor speed is the estimated rotor flux linkage ($\hat{\psi }$), updated according to the following:

(12) $$\hat{\psi }\left(k\right)={\hat{\psi }}^{*}\left(k-1\right)+2\;\eta_{\psi }\left(k\right)\;\omega_{r}\left(k\right)\left(v_{q}\left(k\right)-{\hat{v}}_{q}\left(k\right)\right),$$

with $\eta_{\psi }$ as learning rate. ${\hat{v}}_{q}$ is the estimated q-axis voltage, expressed as follows:

(13) $${\hat{v}}_{q}\left(k\right)={\hat{R}}_{s}^{*}\left(k\right)i_{q}\left(k\right)+\omega_{r}\left(k\right){\hat{\psi }}^{*}\left(k-1\right).$$

According to Appendix B, to ensure the convergence of the flux estimation, the learning rate is computed with the following formula:

(14) $$\eta_{\psi }\left(k\right)=\frac{1-k_{\psi }}{2\omega_{r}{\left(k\right)}^{2}}$$

where $k_{\psi }$ is a real constant in the interval [−1, 1]. Note that, as in Algorithm A3, if AdNN1 was active in the previous step, then ${\hat{\psi }}^{*}\left(k-1\right)$ coincides with $\hat{\psi }\left(k-1\right)$ in (11) providing the following [9],

(15) $$\hat{\psi }\left(k\right)=\hat{\psi }\left(k-1\right)+2\;\eta_{\psi }\left(k\right)\;\omega_{r}\left(k\right)\left(v_{q}\left(k\right)-{\hat{v}}_{q}\left(k\right)\right),$$

otherwise, ${\hat{\psi }}^{*}\left(k-1\right)$ coincides with ${\hat{\psi }}_{stop}\left(x\right)$, allowing the AdNN1 to restart with the best rotor flux linkage estimation available. This reduces the AdNNs transients since they are not reset to their initial values. The estimated rotor flux linkage coincides with the output signal of AdNN1 in Figure 1. 

The AdNN2 shares the same inputs of the AdNN1. The weight of the q-axis current is the estimated stator resistance (${\hat{R}}_{s}$), updated according to the following:

(16) $${\hat{R}}_{s}\left(k\right)={\hat{R}}_{s}^{*}\left(k-1\right)+2\;\eta_{R_{s}}\left(k\right)\;i_{q}\left(k\right)\left(v_{q}\left(k\right)-{\hat{v}}_{q}\left(k\right)\right),$$

with $\eta_{R_{s}}$ as learning rate. ${\hat{v}}_{q}$ is the estimated q-axis voltage, expressed as follows:

(17) $${\hat{v}}_{q}\left(k\right)={\hat{R}}_{s}^{*}\left(k-1\right)i_{q}\left(k\right)+\omega_{r}\left(k\right)\;{\hat{\psi }}^{*}\left(k\right).$$

According to Appendix B, to ensure the convergence of the resistance estimation, the learning rate is computed with the following formula:

(18) $$\eta_{R_{s}}\left(k\right)=\frac{1-k_{R_{s}}}{2i_{q}{\left(k\right)}^{2}}$$

where $k_{R_{s}}$ is a real constant in the interval [−1, 1]. As for the AdNN1, ${\hat{R}}_{s}^{*}\left(k-1\right)$ is replaced with ${\hat{R}}_{s}\left(k-1\right)$ in (14) if AdNN2 was active in the previous step, as follows [9]

(19) $${\hat{R}}_{s}\left(k\right)={\hat{R}}_{s}\left(k-1\right)+2\;\eta_{R_{s}}\left(k\right)\;i_{q}\left(k\right)\left(v_{q}\left(k\right)-{\hat{v}}_{q}\left(k\right)\right).$$

The estimated stator resistance coincides with the output signal of AdNN2 in Figure 1.

The AdNN3 is used for the stator inductance’s estimation. It receives as input the measurements $i_{q}$, $\omega_{r}$, and $v_{d}$, and the signal ${\hat{L}}_{s}^{*}$ from est_star. The weight of the product of the electrical rotor speed and q-axis current is the estimated stator inductance (${\hat{L}}_{s}$), updated according to the following:

(20) $${\hat{L}}_{s}\left(k\right)={\hat{L}}_{s}^{*}\left(k-1\right)+2\;\eta_{L_{s}}\left(k\right)\omega_{r}\left(k\right)i_{q}\left(k\right)\left(v_{d}\left(k\right)-{\hat{v}}_{d}\left(k\right)\right),$$

with $\eta_{L_{s}}$ as learning rate and ${\hat{v}}_{d}$ as the estimated d-axis voltage, expressed as follows:

(21) $${\hat{v}}_{d}\left(k\right)=-\omega_{r}\left(k\right)i_{q}\left(k\right){\hat{L}}_{s}^{*}\left(k-1\right).$$

According to Appendix B, to ensure the convergence of the resistance estimation, the learning rate is computed with the following formula:

(22) $$\eta_{L_{s}}\left(k\right)=\frac{1-k_{L_{s}}}{2i_{q}{\left(k\right)}^{2}}$$

where $k_{L_{s}}$ is a real constant in the interval [−1, 1]. If AdNN3 at the previous step was active, then ${\hat{L}}_{s}\left(k-1\right)$ is used in place of ${\hat{L}}_{s}^{*}\left(k-1\right)$ in (17) as follows [9]

(23) $${\hat{L}}_{s}\left(k\right)={\hat{L}}_{s}\left(k-1\right)+2\;\eta_{L_{s}}\left(k\right)\;\omega_{r}\left(k\right)i_{q}\left(k\right)\left(v_{d}\left(k\right)-{\hat{v}}_{d}\left(k\right)\right).$$

The estimated stator inductance ${\hat{L}}_{s}$ coincides with the output signal of AdNN3 in Figure 1. The values of $k_{\psi }$, $k_{R_{s}},$ and $k_{L_{s}}$ affect the convergence speed of the AdNNs. We recommend choosing these values in the interval [0.8, 1] to mitigate the perturbations of the estimations which occur when the SPMSM changes its operating condition. 

### 3.6. MovAvOut: Moving Average of Measurement Data

The MovAvOut block shown in Figure 1 performs a moving average of the est_star variables. The function calculates, using (24)–(26), the following values:

(24) $${\hat{\psi }}_{ave}^{*}\left(k\right)=\frac{1}{N}\overset{k}{\underset{j=k-N}{\sum }}{\hat{\psi }}^{*}\left(j\right),$$

(25) $${\hat{R}}_{s,ave}^{*}\left(k\right)=\frac{1}{N}\overset{k}{\underset{j=k-N}{\sum }}{\hat{R}}_{s}^{*}\left(j\right),$$

(26) $${\hat{L}}_{s,ave}^{*}\left(k\right)=\frac{1}{N}\overset{k}{\underset{j=k-N}{\sum }}{\hat{L}}_{s}^{*}\left(j\right),$$

where ${\hat{\psi }}_{ave}^{*}$, ${\hat{R}}_{s,ave}^{*}$ and ${\hat{L}}_{s,ave}^{*}$ (gathered in signal ma_out in Figure 1) are the averages of the est_star variables. Note that, as shown in Algorithm A3, the est_star variables coincide with the output of the AdNNs when these ones are enabled by the DMA. Therefore, the MovAvOut block performs a filtering of the estimations produced by the AdNNs. This operation is essential since it allows one to mitigate the effect of the delay of RStatIn and to properly perform the R-statistic algorithm on the parameter estimations.

### 3.7. RStatOut: Identificator of Steady-State Conditions for Estimated Parameters

The RStatOut block in Figure 1 operates in the same way as RStatIn but on different datasets. Its inputs are the ma_out variables and the outputs are computed using (27)–(29):

(27) $$R_{{\hat{\psi }}^{*}{}_{ave}}\left(k\right)=2\frac{\sum_{j=k-N}^{k}{\hat{\psi }}_{n}^{*}{\left(j\right)}^{2}-\frac{1}{N}{\left(\sum_{j=k-N}^{k}{\hat{\psi }}_{n}^{*}\left(j\right)\right)}^{2}}{\sum_{i=k-N+1}^{k}{\left({\hat{\psi }}_{n}^{*}\left(j\right)-{\hat{\psi }}_{n}^{*}\left(j-1\right)\right)}^{2}},$$

(28) $$R_{{\hat{R}}_{s,ave}^{*}}\left(k\right)=2\frac{\sum_{j=k-N}^{k}{\hat{R}}_{s,n}^{*}{\left(j\right)}^{2}-\frac{1}{N}{\left(\sum_{j=k-N}^{k}{\hat{R}}_{s,n}^{*}\left(j\right)\right)}^{2}}{\sum_{j=k-N+1}^{k}{\left({\hat{R}}_{s,n}^{*}\left(j\right)-{\hat{R}}_{s,n}^{*}\left(j-1\right)\right)}^{2}},$$

(29) $$R_{{\hat{L}}_{s,ave}^{*}}\left(k\right)=2\frac{\sum_{j=k-N}^{k}{\hat{L}}_{s,n}^{*}{\left(j\right)}^{2}-\frac{1}{N}{\left(\sum_{j=k-N}^{k}{\hat{L}}_{s,n}^{*}\left(j\right)\right)}^{2}}{\sum_{j=k-N+1}^{k}{\left({\hat{L}}_{s,n}^{*}\left(j\right)-{\hat{L}}_{s,n}^{*}\left(j-1\right)\right)}^{2}}.$$

In these formulas $R_{{\hat{\psi }}^{*}{}_{ave}},\;R_{{\hat{R}}_{s,ave}^{*},}$ and ${\hat{L}}_{s,ave}^{*}$ (gathered in signal R_out in Figure 1) are the R-statistic indices and$\;{\hat{\psi }}_{n}^{*}$, ${\hat{R}}_{s,n,}^{*}$ and ${\hat{L}}_{s,n}^{*}$ are the noisy estimations. Furthermore, in this case, a properly tuned Box–Muller noise is introduced as explained in Section 3.2. The parameters N and $R_{crt}$ and the noise standard deviations $\sigma_{{\hat{\psi }}_{ave}^{*}}$, $\sigma_{{\hat{R}}_{s,ave}^{*},}$ and $\sigma_{{\hat{L}}_{s,ave}^{*}}$ are equal to those defined for the RStatIn block. This function block is used to reveal the stationarity of the DMA outputs, which coincide with the outputs of the AdNNs estimations during their operations, as shown in Algorithm A3. As explained above, this operation allows one to accept only the AdNNs stationary estimations, avoiding the introduction of estimation errors due to transients.

## 4. Hardware-in-the-Loop (HIL) Setup

**Figure 4:**
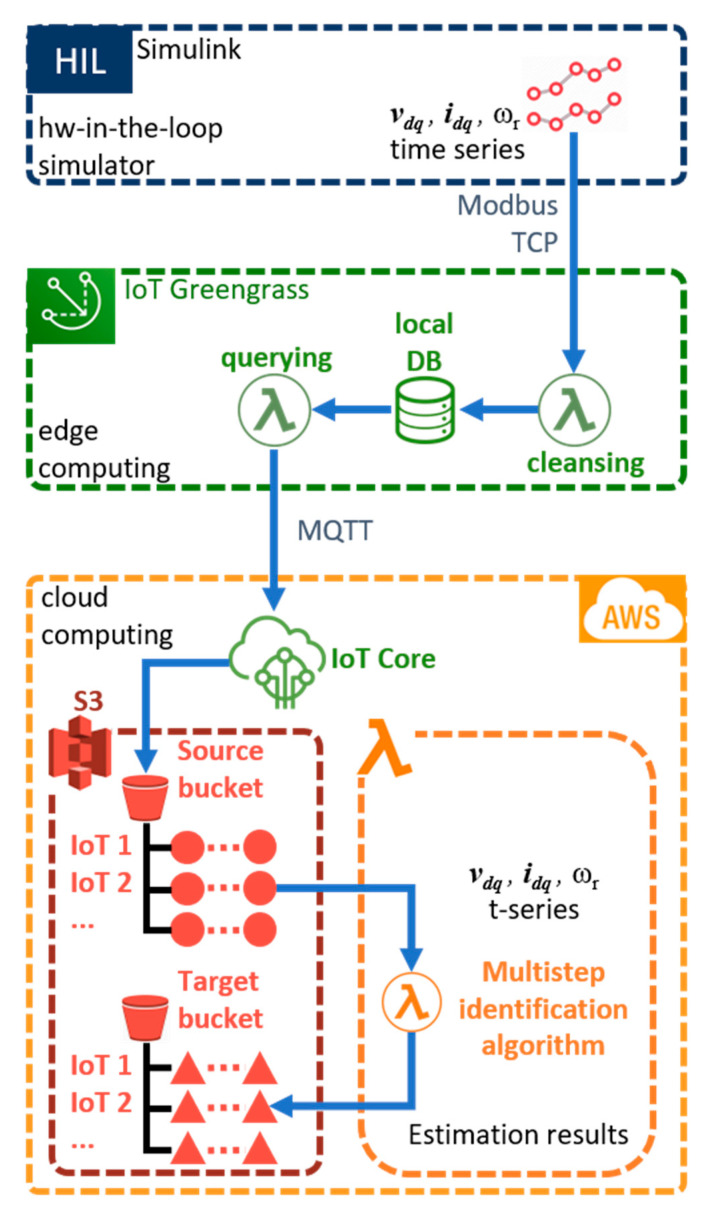
Multistep identification algorithm implemented in an AWS-based cloud prototype.

To evaluate the performance of the proposed solution, the experimental setup in Figure 4 has been arranged. This experimental setup has been inspired by the one shown in [26]. It consists of three levels:

1. A Simulink PC;
2. Internet of things (IoT) devices which elaborate and buffer the collected data in order to optimize the bandwidth towards the cloud;
3. A cloud application where the data is stored and the multistep parameter identification algorithm is executed.

The Simulink PC acts as HIL simulator of the FOC SPMSM. An SPMSM driven by an FOC controller and fed by a pulse-width-modulation (PWM) three-phase inverter has been modeled. PWM is a widely adopted technique for the regulation of power electronic devices such as AC voltage regulators, inverters, rectifiers, etc. [28,29,30]. The FOC controller performs a speed control with zero d-axis current and is made by the cascade of speed and current PI regulators. To bring the model as close to reality as possible, measurement uncertainties are also added to the phase currents and on the DC-link voltage measurements. These uncertainties have been introduced considering datasheets of commercial transducers, i.e., LEM LA 55-P for the currents transducer and LEM LV 25–1000 for the voltage transducer. Moreover, a resolver has been modeled to provide rotor speed and angular position measurements. The SPMSM models with wye-wound stator are configured as Bonfiglioli BMD 400V 65 and 170 surface magnet motors. The main motors and inverter parameters are constant during the simulation and they are summarized in Table 1. The model is used to simulate, at fixed sampling time step, two working cycles of six seconds with variable speed and load torque as shown in the next paragraph. 

The signals $v_{dq}$, $i_{dq,}$ and $\omega_{r}$ are logged, buffered, and sent to the IoT device via Modbus TCP protocol. The IoT architecture has been implemented through the AWS IoT Greengrass technology and performs gathering, pre-cleansing, storing, and analysis operations on the data provided by the HIL simulator [31,32,33]. These operations are performed by two local IoT lambda functions [32], developed using the Python language. The first lambda function fetches and cleans the field data. Then, ready-to-send data are stored in a local database, while the second lambda function queries for the newest data and wraps it in an MQTT [33] message payload which is finally published to the cloud using an MQTT client instance. In this way, the IoT device communicates asynchronously and securely with the cloud over the MQTT protocol using a key pair provisioning mechanism [33]. 

The third level of the setup consists of the cloud application, where messages coming from the IoT device are collected by the AWS IoT Core [34,35]. IoT Core allows one to filter and transform the input messages. An IoT Rule, triggered by an input MQTT message, is implemented to identify, transform, and forward the message payload to Amazon S3. S3 is a hierarchical object storage service in which a source bucket is arranged to receive all the data from the IoT device [36]. The source bucket contains a folder per IoT device, and each folder collects the objects, i.e., the time series generated by the HIL simulator, represented as a red circle in Figure 4. S3 Event Notification is used to trigger the multistep parameter identification every time a new object is saved in the source bucket. The identification algorithm is implemented with a lambda function whose results are recorded in a target bucket, as shown in Figure 4. The tuning parameters of the proposed identification algorithm are reported in Table 2.

**Figure 5:**
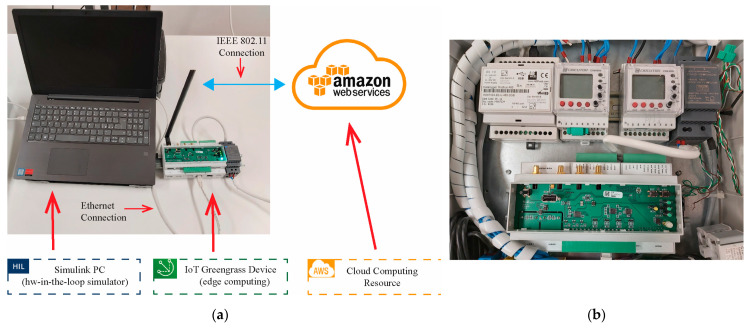
Photo of the experimental setup. (**a**) HIL setup for the validation of the proposed algorithm; (**b**) employed IoT device mounted in an industrial control and monitoring cabinet.

Figure 5a shows a photo of the experimental setup. The IoT device is connected via ethernet cable to the Simulink PC to perform the HIL simulations. The recorded data are sent to the cloud via IEEE 802.11 connection. Note that, even though we employed a simulation environment for the motor data generation, an industrial device has been used as an IoT edge device. Figure 5b shows the IoT device implemented in a real application within an industrial environment interfaced with a data logger and power meters. 

## 5. Simulation Results

**Figure 6:**
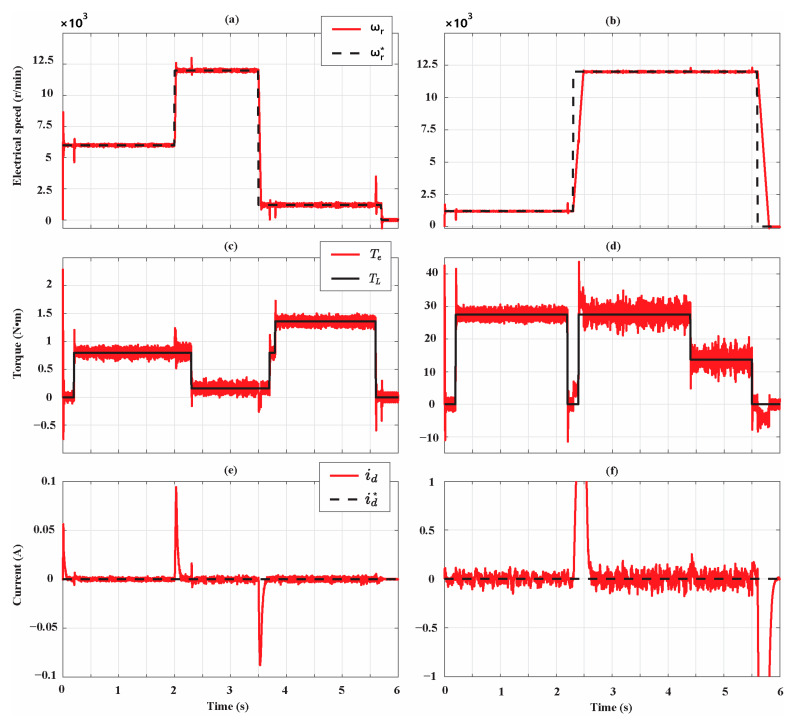
SPMSM working cycles: (**a**,**c**,**e**): speed, torque, and d-axis current profile of the Bonfiglioli 65; (**b**,**d**,**f**): speed, torque, and d-axis current of the Bonfiglioli 170.

Two different working cycles have been simulated for the two motors. The speed, torque, and d-axis current profiles of the simulated working cycles are reported in Figure 6, where $\omega_{r}$, $\omega_{r}{}^{*}$, $T_{e}$, $T_{L}$,$\;i_{d},$ and $i_{d}{}^{*}$ are the measured speed, the reference speed, the electromagnetic torque, the load torque, the measured d-axis current, and the reference d-axis current, respectively. Note that the d-axis current is different from zero only during transients of the SPMSMs. Therefore, it does not affect the performances of the AdNN estimators since they operate only during the steady states of the SPMSMs. We consider two data packets made by 108 s of measurement data recorded from 18 working cycles of the two motors.

**Figure 7:**
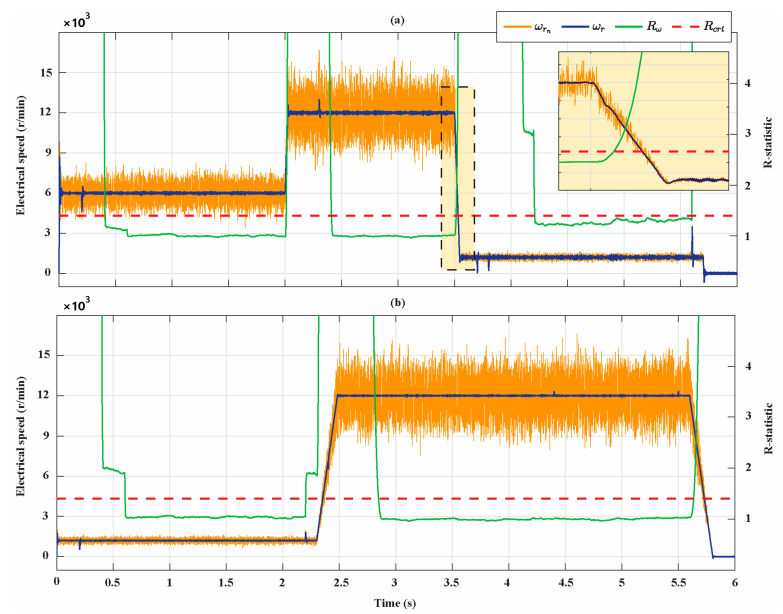
Rotor electrical speed steady-states identification. (**a**) Bonfiglioli 65; (**b**) Bonfiglioli 170.

**Figure 8:**
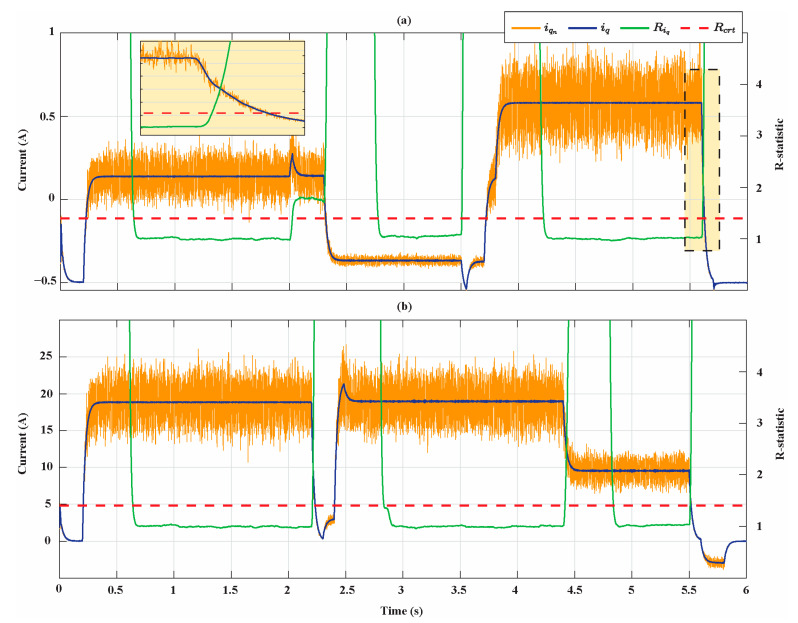
Q-axis current steady-states identification. (**a**) Bonfiglioli 65; (**b**) Bonfiglioli 170.

Figure 7 shows the results of the R-statistic analysis, identifying the rotor speed steady states of the two motors. In this figure, the measured electrical rotor speed $\omega_{r}$, the noisy speed $\omega_{r,n}$ obtained using the Box–Muller noise, the R-statistic values $R_{\omega_{r}},$ and the critical value $R_{crt}$ are shown. Note that the values of $R_{\omega_{r}}\;$and $R_{crt}$ are reported on the right y-axis of the figure. As expected, $R_{\omega_{r}}$ is greater than $R_{crt}$ during transient states, while it is smaller than $R_{crt}$ once the steady state is reached. The R-statistic identifies three rotor speed steady-state time intervals ([0.4 s, 2 s], [2.4 s, 3.5 s], and [4.2 s, 5.6 s]) for the Bonfiglioli 65 and two steady-state time intervals ([0.6 s, 2.2 s], [2.8 s, 5.6 s]) for the Bonfiglioli 170. The zoom in Figure 7a shows how the R-statistic algorithm detects with delay the end of the steady states. Similarly, in Figure 8, the measured q-axis current $i_{q}$, the noisy current $i_{q,n}$, the R-statistic values $R_{i_{q}},$ and the critical value $R_{crt}$ are shown. The R-statistic identifies three q-axis current steady-state time intervals ([0.7 s, 2 s], [2.8 s, 3.5 s], and [4.2 s, 5.6 s]) for the Bonfiglioli 65 and three steady-state time intervals ([0.7 s, 2.2 s], [2.9 s, 4.4 s], and [4.8 s, 5.5 s]) for the Bonfiglioli 170. Moreover, in this case, the zoom in Figure 8a shows how the R-statistic algorithm detects with delay the end of the steady state. Therefore, we deduce that the detected steady-state operating conditions of the two motors are in the time intervals [0.7 s, 2 s], [2.8 s, 3.5 s], and [4.2 s, 5.6 s] for the Bonfiglioli 65 and in the time intervals [0.7 s, 2.2 s], [2.9 s, 4.4 s], and [4.8 s, 5.6 s] for the Bonfiglioli 170.

**Figure 9:**
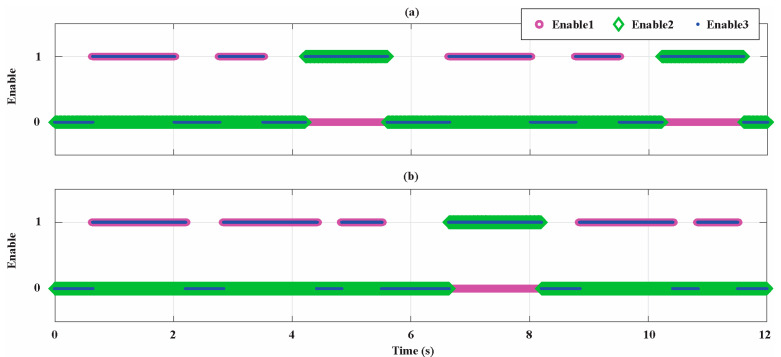
Activation signals for the AdNNs. (**a**) Bonfiglioli 65; (**b**) Bonfiglioli 170.

Figure 9 shows the AdNNs activation signals Enable1, Enable2, and Enable3 in the first 12 s for the two motors. It is shown in which of the motors’ steady states the rotor flux linkage, stator resistance, and stator inductance’s AdNN estimators are working. In the first working cycle of the Bonfiglioli 65, the AdNN1 is activated for the first time at half of the rated speed and current and for the second time at the rated speed and at 10% of the rated current; instead, the AdNN2 is activated only at the 10% of rated speed and at the rated current. Instead, in the first working cycle of the Bonfiglioli 170, only the AdNN1 is activated: the first time at 10% of the rated speed and at the rated current, the second time at the rated speed and current, and the third time at the rated speed and half of the rated current. In both cases, the first activation of AdNN1 corresponds to the first estimation to initialize the multistep algorithm while the other two activations satisfy the other conditions expressed in Algorithm A1. The trend of the enable signals is periodical from 6s to the end of the parameter identification, i.e., the time instant in which the stop criterion is satisfied. In particular, for the Bonfiglioli 170, after 6s, the AdNN1 is activated twice during each working cycle: the first time at the rated speed and current and the second time at the rated speed and half of the rated current. Instead, the AdNN2 is activated only once during each working cycle at the 10% of rated speed and at the rated current. The comparison of the operating conditions in which AdNN1 and AdNN2 are enabled shows how the algorithm is working in agreement with convergence condition in (A13). 

**Figure 10:**
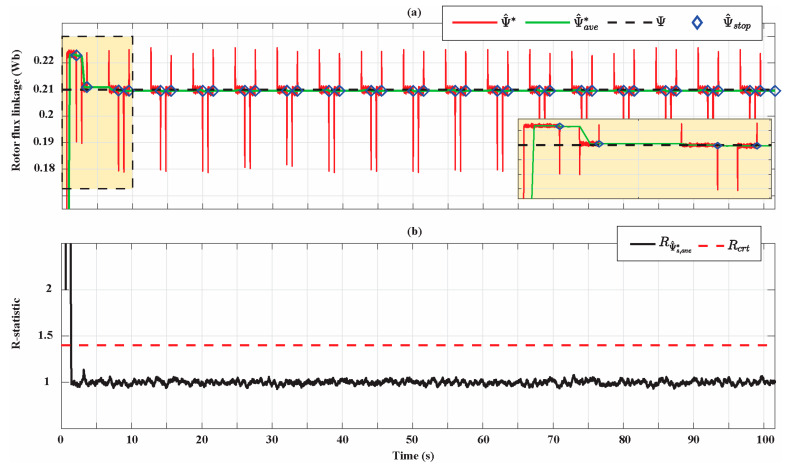
(**a**) Rotor flux linkage estimation for the Bonfiglioli 65; (**b**) R-statistic analysis of the rotor flux linkage estimation.

Figure 10 shows the results of the rotor flux linkage identification for the Bonfiglioli 65. The estimations are progressively improved and the percentage estimation error at the end of the identification process is 0.23%. This figure shows how ${\hat{\psi }}_{\;}{}_{stop}$ changes its value only when the AdNN1 is disabled and is equal to ${\hat{\psi }}^{*}{}_{ave}$ at the end of the AdNN1 operation. In particular, the zoomed part in Figure 10a shows that ${\hat{\psi }}^{*}$ is affected by perturbations before the AdNN1 is disabled, i.e., when ${\hat{\psi }}_{\;}{}_{stop}$ is updated, while the signal ${\hat{\psi }}^{*}{}_{ave}$ overcomes this issue thanks to the filtering operation of the moving average. Note that a progressive improvement of the estimations is achieved in the first two working cycles since the condition expressed by (A13) is satisfied. At the third working cycle, an accurate estimation has already been achieved and there are no substantial improvements until the stop criterion is satisfied. Figure 10b shows that R-statistic manages to detect only the first transient state of ${\hat{\psi }}^{*}{}_{ave}$, while the other transients are too small to be detected. The comparison between Figure 10a,b shows how all the estimations performed by the AdNN1 have reached the steady state and, thus, have been processed by the DMA to update ${\hat{\psi }}_{\;}{}_{stop}$. 

**Figure 11:**
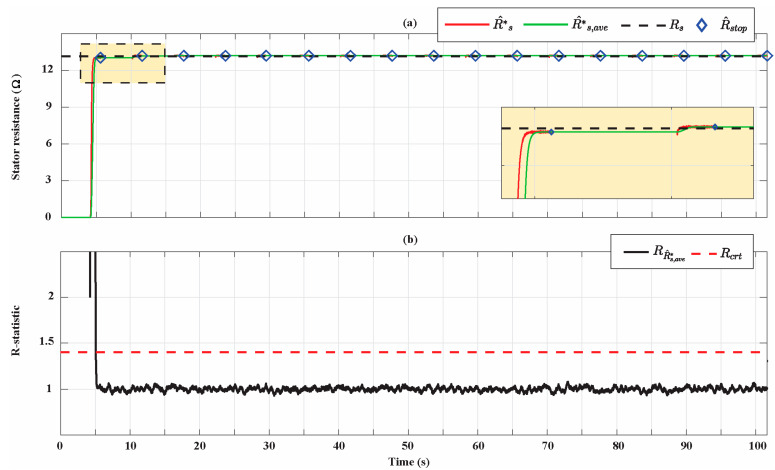
(**a**) Stator resistance estimation for the Bonfiglioli 65; (**b**) R-statistic analysis of the stator resistance estimation.

Figure 11 shows the results of the stator resistance identification for the Bonfiglioli 65. Moreover, in this case, there is a progressive improvement of the estimations and at the end of the identification process a percentage estimation error of 0.35% is achieved. Similar considerations already performed for the flux linkage estimations can be repeated for the updating of ${\hat{R}}_{stop}$ and the R-statistic analysis. 

**Figure 12:**
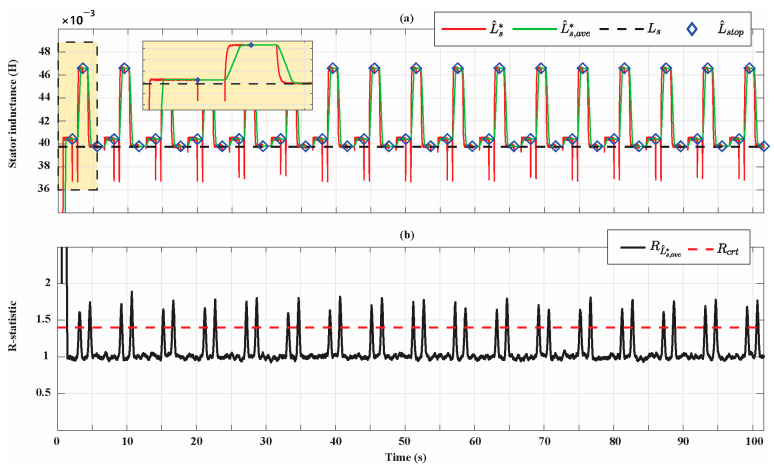
(**a**) Stator inductance estimation for the Bonfiglioli 65; (**b**) R-statistic analysis of the stator inductance estimation.

**Figure 13:**
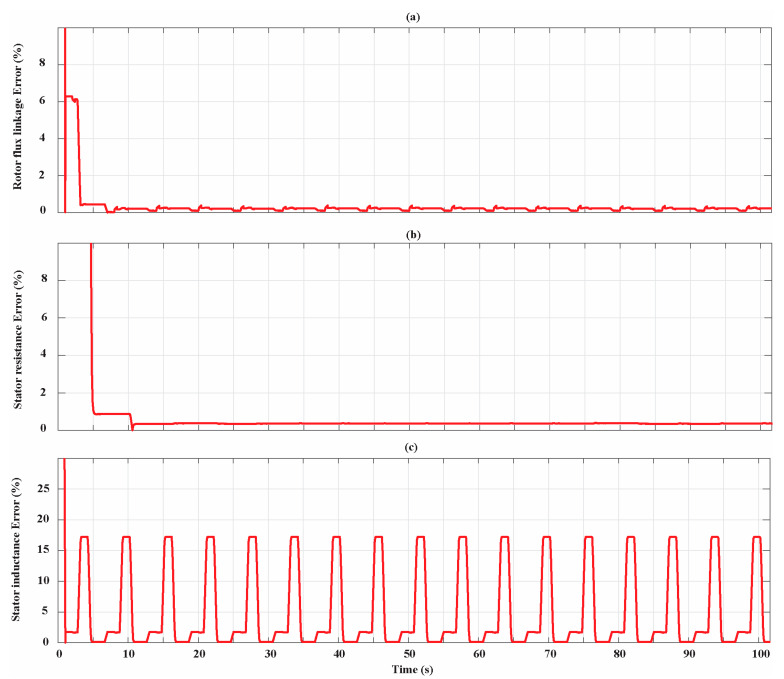
Percentage estimation error for the Bonfiglioli 65. (**a**) Rotor flux linkage estimation error; (**b**) stator resistance estimation error; (**c**) stator inductance estimation error.

Figure 12 shows how the stator inductance estimation accuracy slightly depends on the SPMSM operating condition in which the AdNN3 is activated. The condition in which the minimum estimation error is achieved is the one at the rated current and 10% of the rated speed while the condition in which the maximum error is achieved is the one at 10% of the rated current and at the rated speed. At the end of the parameter identification process, the relative percentage estimation error is 0.11%. Moreover, in this case, the figure shows the importance of filtering the AdNN estimation with the MovAvOut block in order to attenuate perturbations that affect the estimation accuracy. Figure 12b shows that, in this case, R-statistic managed to detect the transients of the estimations. However, all the estimations performed by the AdNN3 reached the steady state and have been processed by the DMA to update ${\hat{L}}_{stop}$. The percentage estimation errors associated with the filtered parameter estimations ${\hat{\psi }}_{ave}^{*}$, ${\hat{R}}_{s,ave,}^{*}$ and ${\hat{L}}_{s,ave}^{*}$ for the entire simulation time of the Bonfiglioli 65 are shown in Figure 13. 

**Figure 14:**
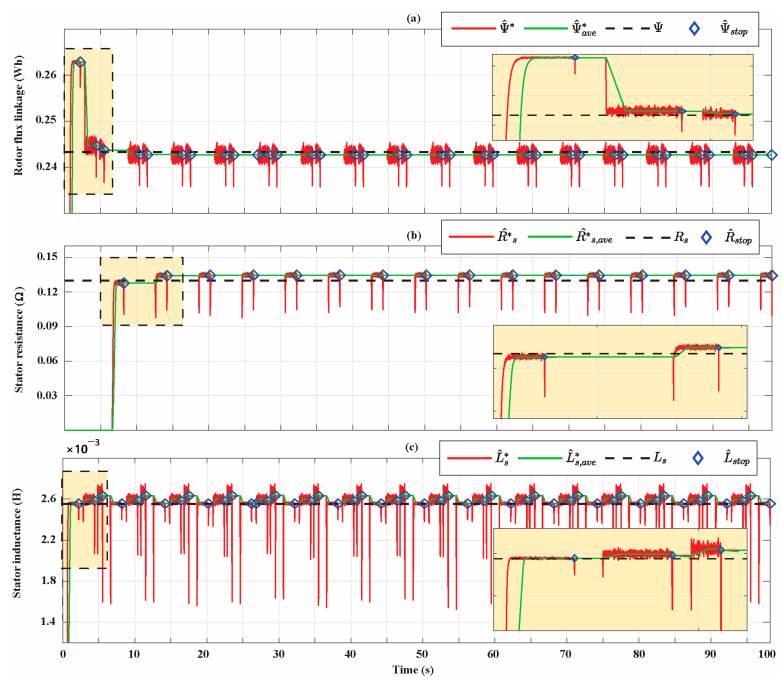
(**a**) Rotor flux linkage estimation for the Bonfiglioli 170; (**b**) stator resistance estimation for the Bonfiglioli 170; (**c**) stator inductance estimation for the Bonfiglioli 170.

**Figure 15:**
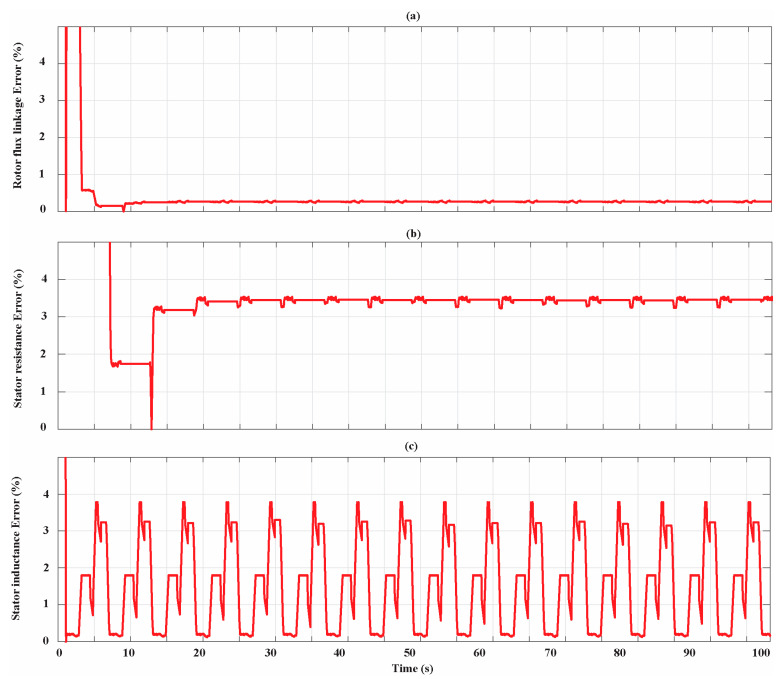
Percentage estimation error for the Bonfiglioli 170. (**a**) Rotor flux linkage estimation error; (**b**) stator resistance estimation error; (**c**) stator inductance estimation error.

Figure 14 shows the parameters identification for the Bonfiglioli 170. The estimations of rotor flux linkage, stator resistance, and inductance have trends similar to those of Bonfiglioli 65. In particular, the rotor flux linkage and stator resistance estimation progressively improve and the percentage estimation errors at the end of the identification process are 0.27% and 3.18%, respectively. Moreover, in this case, the stator inductance estimation accuracy depends on the operating conditions of the SPMSM in which the AdNN3 is activated. The condition in which the minimum estimation error is achieved is the one at the rated current and 10% of the rated speed while the condition in which the maximum error is achieved is the one at 50% of the rated current and at the rated speed. At the end of the parameter identification process, the percentage estimation error is 0.16%. The percentage estimation errors associated with the filtered parameter estimations ${\hat{\psi }}_{ave}^{*}$, ${\hat{R}}_{s,ave,}^{*}$ and ${\hat{L}}_{s,ave}^{*}$ for the entire simulation time Bonfiglioli 170 are shown in Figure 15. 

Finally, the algorithm runs on a cloud resource with 8 Intel Xeon Platinum 8259CL @ 2.50 GHz processors and 32 GB of RAM. Execution timings of multiple parallel runs of the identification procedure of the Bonfiglioli 170 are reported in Table 3. Note that the total execution time highlights the computational sustainability of the proposed scheme in large-scale applications using cloud computing resources. The achieved values are appropriate since for parallel runs less than the number of cores, the execution times are similar, i.e., approximately one core per run is dedicated. Instead, for parallel runs greater than the number of cores, the execution time proportionally increases as the number of parallel runs increases. 

## 6. Conclusions

In this paper, a new multistep multiparametric identification method of SPMSMs highly suitable for cloud computing environment is presented. The results achieved show how the proposed solution overcame the rank deficiency problem without any signal injection and extra sensor, with the combined action of the stator inductance, resistance, and rotor PM flux linkage estimations. The R-static algorithm managed to identify the steady states of the SPMSM and of the parameter estimations. Moreover, the results of the parameter estimations confirm the validity of the convergence condition obtained by means of analytical studies and employed in the design of the DMA. We achieved final estimation errors of 0.23%, 0.35%, and 0.11% for the rotor flux linkage, stator resistance, and stator inductance, respectively, in a simulation environment. Finally, the criteria proposed for the automated tuning of the Box–Muller noise and of the AdNNs learning rates have also been validated by the achieved results. 

We stress that the proposed method is highly suitable for the cloud computing implementation in large-scale applications since it requires a simple tuning of a few parameters. Moreover, note that the proposed algorithm can be partially reused for applications other than PMSMs. In particular, steady-state identification procedures of the motor and the estimated parameters can also be implemented for other motor typologies. Instead, the AdNNs and the convergence condition need to be modified since they are based on the mathematical model of the motor to be identified. 

Future developments will include tests with motor data provided by real production plants, and the extension of the method to different machines, such as internal PMSMs, will be considered. 

## Figures and Tables

**Table 1:** Motor and inverter parameters.

Parameters	Bonfiglioli 65	Bonfiglioli 170
Rated power (kW)	0.5	8.6
Rated current (A)	1.33	18.6
Rated torque (N·m)	1.6	27.5
Rated speed (r/min)	3000	3000
Number of pole pairs	4	4
$R_{s}$ (Ω)	13.1550	0.13
$L_{s}$ (mH)	39.75	2.55
ψ (Wb)	0.21	0.2433
$K_{c\;}$ (N·m/A)	1.26	1.46
$J\;\left(\mathrm{Kg}\cdot m^{2}\right)$	$0.04\times 10^{-3}$	$2.82\times 10^{-3}$
Switching frequency (Hz)	$50\times 10^{4}$	$50\times 10^{4}$
DC-link voltage (V)	720	720
Sample time (s)	$2\times 10^{-4}$	$2\times 10^{-4}$
Phase current uncertainty (%)	±0.65	±0.65
DC voltage uncertainty (%)	±0.8	±0.8

**Table 2:** Parameters of the proposed algorithm.

**Parameters**	Value
N	2000
$R_{crt}$	1.4
$\sigma_{\omega_{r}}\left(k\right)$	$10\%\omega_{r}\left(k\right)$
$\sigma_{i_{q}}\left(k\right)$	$10\%i_{q}\left(k\right)$
$\sigma_{{\hat{\psi }}_{ave}^{*}}\left(k\right)$	$10\%{\hat{\psi }}_{ave}^{*}\left(k\right)$
$\sigma_{{\hat{R}}_{s,ave}^{*}}\left(k\right)$	$10\%{\hat{R}}_{s,ave}^{*}\left(k\right)$
$\sigma_{L_{s,ave}^{*}}\left(k\right)$	$10\%{\hat{L}}_{s,ave}^{*}\left(k\right)$

**Table 3:** Execution times of multiple parallel runs.

Number of Parallel Runs	Total Execution Time
2	3 m 10 s
4	3 m 7 s
8	4 m 48 s
16	9 m 21 s
32	19 m 5 s
64	38 m 30 s

## Data Availability

The data presented in this study are available on request from the corresponding author. The data are not publicly available due to privacy issues.

## Appendix A

**Algorithm A1.** Operating condition check and AdNNs enable.
1. **if** x=0 2. Enable1(k)=ON, Enable2(k)=OFF, Enable3(k)=ON 3. **else if** $\left(\omega_{rR}/\omega_{r,ave}\left(k\right)\right)\cdot \left(i_{q,ave}\left(k\right)/i_{qR}\right)<0.95$ **OR** $(\omega_{r,ave}\left(k\right)/\omega_{r\psi })\cdot \left(i_{q\psi }/i_{q,ave}\left(k\right)\right)>0.95$ 4. Enable1(k)=ON, Enable2(k)=OFF, Enable3(k)=ON 5. **else if** $(\omega_{r,ave}\left(k\right)/\omega_{r\psi })\cdot \left(i_{q\psi }/i_{q,ave}\left(k\right)\right)<0.95\;$ 6. Enable1(k)=OFF, Enable2(k)=ON, Enable3(k)=ON 7. **else** 8. Enable1(k)=OFF, Enable2(k)=OFF, Enable3(k)=OFF 9. **end**

**Algorithm A2.** Estimations steady-state check and partial estimations update.
1. Enable1(k)=OFF, Enable2(k)=OFF, Enable3(k)=OFF 2. **if** $R_{{\hat{\psi }}_{ave}^{*}}\left(k-1\right)<R_{crt\;}\;$**AND** Enable1(k−1)=ON 3. x=x+1 4. ${\hat{\psi }}_{stop}\left(x\right)={\hat{\psi }}^{*}{}_{ave}\left(k-1\right)$ 5. $\omega_{r\psi }\left(x\right)=\omega_{r,ave}\left(k-1\right)$, $i_{q\psi }\left(x\right)=i_{q,ave}\left(k-1\right)$ 6. **end** 7. **if** $R_{{\hat{R}}_{s,ave}^{*}}\left(k-1\right)<R_{crt\;}$ **AND** Enable2(k−1)=ON 8. y=y+1 9. ${\hat{R}}_{stop}\left(y\right)={\hat{R}}^{*}{}_{s,ave}\left(k-1\right)$ 10. $\omega_{rR}\left(y\right)=\omega_{r,ave}\left(k-1\right)$, $i_{qR}\left(y\right)=i_{q,ave}\left(k-1\right)$ 11. **end** 12. **if** $R_{{\hat{L}}_{s,ave}^{*}}\left(k-1\right)<R_{crt\;}\;$ **AND** Enable3(k−1)=ON 13. z=z+1 14. ${\hat{L}}_{stop}\left(z\right)={\hat{L}}^{*}{}_{s,ave}\left(k-1\right)$ 15. **end**

**Algorithm A3.** Update est_star.
1. **if** Enable1(k)=ON 2. ${\hat{\psi }}^{*}\left(k\right)=\hat{\psi }\left(k\right),\;\;{\hat{R}}_{s}^{*}\left(k\right)={\hat{R}}_{stop}\left(y\right),\;\;{\hat{L}}_{s}^{*}\left(k\right)=\hat{L}\left(k\right)$ 3. **else if** Enable2(k)=ON 4. ${\hat{\psi }}^{*}\left(k\right)={\hat{\psi }}_{stop}\left(x\right),\;\;{\hat{R}}_{s}^{*}\left(k\right)=\hat{R}\left(k\right),\;\;{\hat{L}}_{s}^{*}\left(k\right)=\hat{L}\left(k\right)$ 5. **else** 6. ${\hat{\psi }}^{*}\left(k\right)={\hat{\psi }}_{stop}\left(x\right),\;\;{\hat{R}}_{s}^{*}\left(k\right)={\hat{R}}_{stop}\left(y\right),\;\;{\hat{L}}_{s}^{*}\left(k\right)={\hat{L}}_{stop}\left(z\right)$

## Appendix B

In this appendix we prove the convergence condition (11).

Substituting (13) in (14) and assuming it to be in steady-state conditions, the following first order difference equation is obtained:

(A1) $$\hat{\psi }\left(k\right)=\hat{\psi }\left(k-1\right)\left(1-2\;\eta_{\psi }\left(k\right)\omega_{r}{}^{2}\right)+2\;\eta_{\psi }\left(k\right)\;\omega_{r}\left(v_{q}-{\hat{R^{*}}}_{s}i_{q}\right),$$

where $\omega_{r}=\omega_{r\psi }$, $v_{q}=v_{q\psi }$, $i_{q}=i_{q\psi }$ are steady values. Note that in this case ${\hat{R^{*}}}_{s}={\hat{R}}_{\;}{}_{stop}$ is constant during the operation of the AdNN1. The solution of Equation (A1) is the sum of the solution of the homogeneous equation plus a particular solution. Therefore, all solutions of (A1) are in the following form:

(A2) $$\hat{\psi }\left(k\right)=c{\left(1-2\;\eta_{\psi }\left(k\right)\;\omega_{r\psi }{}^{2}\right)}^{k}+\frac{v_{q\psi }-{\hat{R}}_{\;}{}_{stop}i_{q\psi }}{\omega_{r\psi }},$$

with c ∈ℝ. $\eta_{\psi }$ is chosen to make $\left|1-2\;\eta_{\psi }\left(k\right)\;\omega_{r\psi }{}^{2}\right|<1.$ Therefore, in steady-state conditions, for k→∞ the estimated rotor flux linkage is:

(A3) $${\hat{\psi }}_{\infty }=\frac{v_{q\psi }-{\hat{R}}_{\;}{}_{stop}i_{q\psi }}{\omega_{r\psi }}.$$

Defining ${\hat{R}}_{\;}{}_{stop}=R_{s}+\varepsilon_{R}$, with $R_{s}$ as the actual stator resistance and $\varepsilon_{R}$ as the stator resistance estimation error and substituting in (A3), we obtain:

(A4) $${\hat{\psi }}_{\infty }=\frac{v_{q\psi }-R_{s}i_{q\psi }}{\;\omega_{r\psi }}+\frac{i_{q\psi }}{\omega_{r\psi }}\varepsilon_{R}.$$

Neglecting measurement errors and considering (4), we deduce that the first term of (A4) is equal to the actual rotor flux linkage:

(A5) $$\psi =\frac{v_{q\psi }-R_{s}i_{q\psi }}{\;\omega_{r\psi }}.$$

Therefore, the second term of (A4) is the rotor flux linkage estimation error:

(A6) $$\varepsilon_{\psi }=\frac{i_{q\psi }}{\omega_{r\psi }}\varepsilon_{R}.$$

Thus, (A4) can be rewritten as:

(A7) $${\hat{\psi }}_{\infty }=\psi +\varepsilon_{\psi }.$$

Note that, from (A6), the flux linkage estimation error can be minimized if the AdNN1 operates with high values of $\omega_{r}$ and low values of $i_{q}$.

Repeating the same steps for the stator resistance, we obtain

(A8) $${\hat{R}}_{s\infty }=\frac{v_{qR}-\omega_{rR}\psi }{\;i_{qR}}+\frac{\omega_{rR}}{\;i_{qR}}\varepsilon_{\psi }=R_{s}+\varepsilon_{R}.$$

Since ${\hat{\psi }}_{\;}{}_{stop}$ and ${\hat{R}}_{\;}{}_{stop}$ are updated by the DMA only in the steady-state conditions of the estimations, we can assume that ${\hat{\psi }}_{\;}{}_{stop}\approx {\hat{\psi }}_{\infty }$ and ${\hat{R}}_{\;}{}_{stop}\approx {\hat{R}}_{s\infty }$. Let us consider that resistance and rotor flux estimations are provided alternatively. Therefore, considering the j-th steady state in which the AdNN1 is enabled, the following equation is obtained according to (A4) and (A7):

(A9) $${\hat{\psi }}_{\;}{}_{stop}\left(j\right)=\frac{v_{q\psi }-R_{s}i_{q\psi }}{\;\omega_{r\psi }}+\frac{i_{q\psi }}{\omega_{r\psi }}\varepsilon_{R}=\psi +\varepsilon_{\psi }\left(j\right).$$

According to (A8), the value of the estimated stator resistance before the j-th steady state is achieved is:

(A10) $${\hat{R}}_{\;}{}_{stop}=\frac{v_{qR}-\omega_{rR}\psi }{\;i_{qR}}+\frac{\omega_{rR}}{\;i_{qR}}\varepsilon_{\psi }\left(j-1\right)=R_{s}+\varepsilon_{R}.$$

where $\varepsilon_{\psi }\left(j-1\right)$ is the rotor flux estimation error related to ${\hat{\psi }}_{\;}{}_{stop}\left(j-1\right)$. Substituting $\varepsilon_{R}$ in (A9):

(A11) $$\varepsilon_{\psi }\left(j\right)=\frac{i_{q\psi }}{\omega_{r\psi }}\cdot \frac{\omega_{rR}}{\;i_{qR}}\varepsilon_{\psi }\left(j-1\right).$$

The solution of this equation is:

(A12) $$\varepsilon_{\psi }\left(j\right)=c{\left(\frac{i_{q\psi }}{i_{qR}}\cdot \frac{\omega_{rR}}{\omega_{r\psi }}\right)}^{j}.$$

Therefore, the estimation error asymptotically converges to zero if the following is satisfied:

(A13) $$\frac{i_{q\psi }}{i_{qR}}\cdot \frac{\omega_{rR}}{\omega_{r\psi }}<1.$$

If $\varepsilon_{\psi }$ is zero, then also $\varepsilon_{R}$ will be zero, according to (A6). Therefore, the inequality (11) has been proven.

Inequality (A13) expresses the convergence condition of the whole multistep parameter identification algorithm. The DMA has been designed to lead the AdNNs to operate properly under the SPMSMs operating conditions which satisfy (A13). It is clear that the proposed method can be applied only if the SPMSM operates in different operating conditions that make it possible to satisfy (A13).

## Appendix C

In this appendix, we prove how the learning rate of the AdNNs expressed by (14), (18) and (22) guarantee convergence.

According to (A2), to ensure the convergence of the flux estimations provided by AdNN1, the learning rate $\eta_{\psi }$ should be chosen to make $\left|1-2\;\eta_{\psi }\;\omega_{r}{\left(k\right)}^{2}\right|<1$. Therefore, the following equation must be satisfied:

(A14) $$1-2\;\eta_{\psi }\;\omega_{r}{\left(k\right)}^{2}=k_{\psi },\;\;\;\;k_{\psi }\;\epsilon \;]-1,\;1[.\;$$

The learning rate $\eta_{\psi }$ which satisfies (A14) is:

(A15) $$\;\eta_{\psi }\left(k\right)=\frac{1-k_{\psi }}{2\omega_{r}{\left(k\right)}^{2}},\;\;\;\;k_{\psi }\;\epsilon \;]-1,\;1[.\;$$

The same considerations can be repeated for the AdNN stator resistance and inductance estimators.
